# Utilization of the Pancreas From Donors With an Extremely High Pancreas Donor Risk Index: Report of the National Registry of Pancreas Transplantation

**DOI:** 10.3389/ti.2023.11132

**Published:** 2023-05-17

**Authors:** Keizo Kaku, Yasuhiro Okabe, Shinsuke Kubo, Yu Sato, Takanori Mei, Hiroshi Noguchi, Yoshito Tomimaru, Toshinori Ito, Takashi Kenmochi, Masafumi Nakamura

**Affiliations:** ^1^ Department of Surgery and Oncology, Graduate School of Medical Sciences, Kyushu University, Fukuoka, Japan; ^2^ Department of Gastroenterological Surgery, Graduate School of Medicine, Osaka University, Suita, Japan; ^3^ The Japan Pancreas Transplant Registry, Japan Society for Pancreas and Islet Transplantation, Suita, Japan; ^4^ Osaka Center for Cancer and Cardiovascular Disease Prevention, Osaka, Japan; ^5^ Department of Transplantation and Regenerative Medicine, School of Medicine, Fujita Health University, Toyoake, Japan

**Keywords:** pancreas transplantation, graft survival, type 1 diabetes mellitus, thrombosis, prognostic factor

## Abstract

Pancreas transplants from expanded criteria donors are performed widely in Japan because there is a shortage of brain-dead donors. However, the effectiveness of this strategy is unknown. We retrospectively studied 371 pancreas transplants to evaluate the possibility of pancreas transplantation from expanded criteria donors by the Pancreas Donor Risk Index (PDRI). Patients were divided into five groups according to quintiles of PDRI values (Q1–Q5). The 1-year pancreas graft survival rates were 94.5% for Q1, 91.9% for Q2, 90.5% for Q3, 89.3% for Q4, and 79.6% for Q5, and were significantly lower with a lower PDRI (*p* = 0.04). A multivariate analysis showed that the PDRI, donor hemoglobin A1c values, and pancreas transplantation alone significantly predicted 1-year pancreas graft survival (all *p* < 0.05). Spline curve analysis showed that the PDRI was incrementally associated with an increased risk of 1-year graft failure. In the group with a PDRI ≥ 2.87, 8/56 patients had graft failures within 1 month, and all were due to graft thrombosis. The PDRI is a prognostic factor related to the 1-year graft survival rate. However, pancreas transplantation from high-PDRI donors shows acceptable results and could be an alternative when the donor pool is insufficient.

## Graphical Abstract

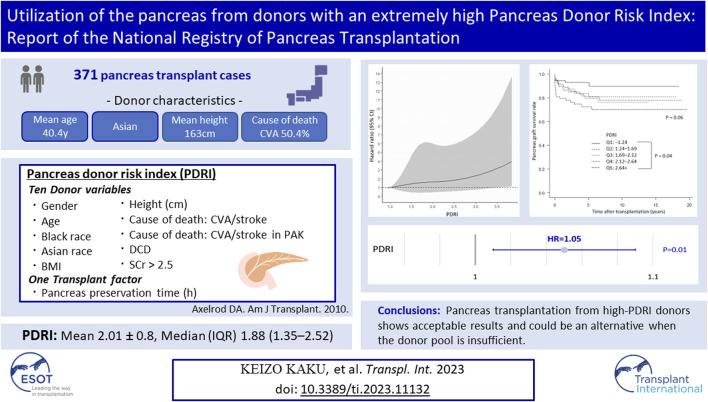

## Introduction

Pancreas transplantation enables insulin withdrawal in patients with insulin-dependent diabetes and considerably improves patients’ survival and quality of life [1–4]. However, in Japan, a shortage of brain-dead donors has resulted in a long waiting period. Pancreas transplantation from expanded criteria donors is widely performed because a prolonged waiting period worsens the prognosis of life [5, 6]. In Japan, the donor age is relatively high, with 43% of donors older than 45 years, and 51% of deaths are due to cerebrovascular accidents [7]. Although pancreas transplants are performed in such a special background with many expanded criteria donors, the results are relatively excellent [7]. However, a major drawback for expanded criteria donors is the lack of objective criteria. In practice, the donor’s eligibility is determined by each facility’s criteria on the basis of a comprehensive evaluation of factors, such as the donor’s age, weight, body mass index (BMI), and hemoglobin A1c (HbA1c) values. Japanese national data analyses have reported that the donor’s age is not associated with the prognosis [8] and that no single donor factor affects the prognosis [9], but which expanded criteria donors are acceptable remain unclear. There are a variety of factors that define an expanded criteria donor; therefore, it should be evaluated using a comprehensive and objective index.

In pancreas transplantation, the Pancreas Donor Risk Index (PDRI), which was reported by Axelrod et al. in 2010, is currently used to predict 1-year pancreas graft survival as a pre-procurement scoring system [10]. The PDRI was created using 10 donor factors and the pancreas preservation time for the US population. The donor factors consist of the following: sex, age, black race, Asian race, BMI, height, cerebrovascular accident (CVA)/stroke, CVA/stroke in pancreas transplantation after kidney transplantation (PAK), donation after circulatory death, and serum creatinine (SCr) concentrations. The PDRI is designed so that the median donor has a Donor Risk Index of 1.0. A higher Donor Risk Index indicates a higher risk of graft failure. An elevated PDRI is associated with an increased 1-year graft failure rate. A review of the reports that have evaluated the PDRI to date showed that the highest value of the PDRI was 3.40 [11]. Additionally, only a relatively narrow range of the PDRI has been used to evaluate the PDRI [12–18].

A high percentage of grafts are discarded because pancreas grafts are often evaluated under relatively strict criteria [19, 20]. In recent years, there has been a trend to make effective use of pancreatic grafts, which have been discarded in the past, for the purpose of effective use of organs. In the absence of other risk factors, deregulating the criteria for BMI and donor age is acceptable [19]. Furthermore, transplantation from a mildly obese donor can be safely performed [21]. In this trend of reregulating donor criteria and increasing transplantation opportunities, Japanese data, which have accumulated a large number of transplant results from expanded criteria donors, are considered to be effective for determining donor indications. This study aimed to evaluate pancreas transplant donors in Japan using the PDRI and to examine the possibility of the effective use of expanded criteria donors.

## Patients and Methods

### Study Population

A total of 400 pancreas transplants performed at 18 certified pancreas transplant centers in Japan between January 2001 and July 2019 were included in this study. Of these, 371 cases were included after excluding 27 cases of living pancreas transplantation and two cases of incomplete data. The primary disease was type 1 diabetes mellitus in all cases.

The following clinical data were retrospectively extracted from the national database administered by the Japan Society for Pancreas and Islet Transplantation: transplantation type, recipient age, recipient sex, recipient height, recipient BMI, duration of type 1 diabetes mellitus, episode of preoperative dialysis, duration of dialysis, donor age, donor sex, donor height, donor BMI, donor HbA1c concentrations, cause of death, episode of cardiopulmonary resuscitation, SCr concentrations, total ischemic time of the pancreas graft, pancreas graft position, ductal management, type of venous drainage, artery reconstruction, gastroduodenal artery reconstruction, and portal vein extension. Written informed consent was obtained for enrollment in the registry of the Japan Society for Pancreas and Islet Transplantation. The application and approval of the institutional review board were exempt because all data and information used in this study were de-identified. This study was conducted in accordance with the principles of the Declaration of Helsinki and Istanbul.

### Study Design

The PDRI of Japanese patients with a pancreas transplant was calculated according to the formula reported by Axelrod et al. [10] Several cutoff values for the PDRI were set, and the short-term pancreas graft survival rate was verified. The short-term graft survival rate was defined as the 1-year graft survival rate. To analyze the long-term prognosis, the 5-year graft and patients’ survival rates were verified. An analysis of prognostic factors related to 1-year pancreas graft survival was performed. The target population was narrowed down to patients with a high PDRI, and the 1-year graft survival rate was verified. Pancreas graft failure was defined as the time when the C-peptide value became <0.3 ng/mL or at the time of graft extraction.

### Statistical Analysis

Categorical variables are presented as the count (percentage) and were compared using Fisher’s exact test or the χ^2^ test, as appropriate. Continuous variables are presented as the mean ± standard deviation and were analyzed using the Mann–Whitney U test. Kaplan–Meier curves with log rank tests were used to examine graft and patients’ survival. Bonferroni correction was used to adjust for multiple comparisons. Potential risk factors for 1-year pancreas graft survival were assessed using univariate and multivariate Cox proportional hazards analyses. Restricted cubic spline curves were plotted to describe the multivariable-adjusted association between the PDRI and the hazard ratio (HR) with the 95% confidence interval (CI) for graft survival. The cutoff value of the PDRI calculated from receiver operating characteristic curve analysis was chosen as the reference for the spline plot. Statistical significance was set at *p* < 0.05. All statistical analyses were performed using JMP 16.0.0 (SAS Institute, Cary, NC), EZR (Easy R) version 1.54 (Saitama Medical Center, Jichi Medical University, Saitama, Japan) [22], and R version 4.1.2 (The R Foundation for Statistical Computing, Vienna, Austria).

## Results

### Distribution of the PDRI

**FIGURE 1:**
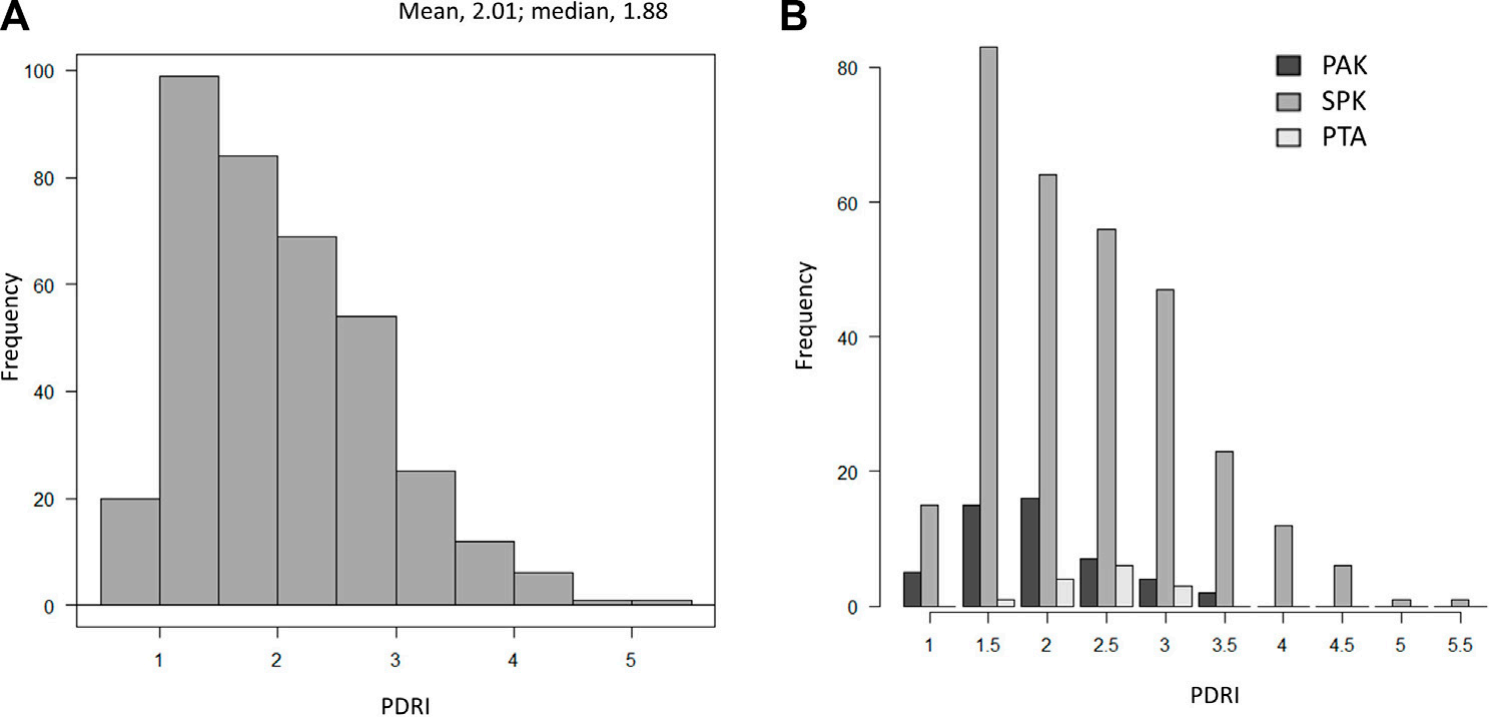
Distribution of PDRI values. **(A)** Distribution of PDRI values in Japan. **(B)** Distribution of PDRI values in Japan by the transplant type. PDRI, pancreas donor risk index; PAK, pancreas transplantation after kidney transplantation; SPK, simultaneous pancreas and kidney transplantation; PTA, pancreas transplantation alone.

The distribution of the mean PDRI of the 371 patients in Japan is shown in Figure 1A. There were 308 simultaneous pancreas kidney transplantations, 49 PAKs, and 14 pancreas transplantations alone (PTAs), and all but three patients who underwent transplantation from cardiac death donors underwent transplantation from brain-dead donors. The mean PDRI was 2.01 ± 0.8 and the median PDRI (interquartile range; IQR) was 1.88 (1.35–2.52). The distribution of the PDRI according to the transplantation type is shown in Figure 1B.

### Pancreas Graft Survival Rate and Patients’ Survival Rate by the PDRI

**FIGURE 2:**
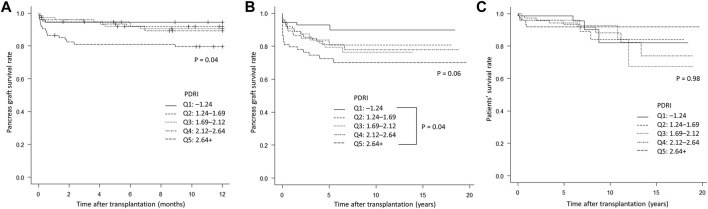
Kaplan–Meier curves comparing the five groups (Q1–Q5) according to quintiles of PDRI values. **(A)** Short-term pancreas graft survival rate. **(B)** Long-term pancreas graft survival rate. **(C)** Patients’ survival rate. PDRI, pancreas donor risk index.

Forty of the 371 patients had pancreas graft failure within 1 year of transplantation. The median (interquartile range) time to pancreas graft failure was 14 days (2.75–113.25 days). The causes of graft were thrombosis in 22 cases, rejection in seven cases, graft duodenal perforation in seven cases, non-adherence in two cases, recurrent type 1 diabetes in one case, and unknown in one case. Patients were divided into five groups (Q1–Q5) according to the quintile of the PDRI value (Table 1). Significant differences in donor age (*p* < 0.001), height (*p* < 0.01), BMI (*p* < 0.001), cause of death (*p* < 0.001), and TIT of the pancreas graft (*p* = 0.01), which are factors that constitute the PDRI, were found between the five groups. Other than the factors constituting the PDRI, significant differences were observed in HbA1c values (*p* < 0.001), cardiopulmonary resuscitation (*p* < 0.01), and graft position (*p* = 0.01) between the groups. The 1-year pancreas graft survival rates were 94.5% for Q1, 91.9% for Q2, 90.5% for Q3, 89.3% for Q4, and 79.6% for Q5, which were significantly lower with a lower PDRI (*p* = 0.04, Figure 2A). With regard to the long-term prognosis, the 5-year pancreas graft survival rates were 92.9% for Q1, 83.7% for Q2, 79.3% for Q3, 81.0% for Q4, and 72.5% Q5. The 5-year pancreas graft survival rate for Q5 was significantly lower than that for Q1 (*p* = 0.04, Figure 2B). The 5-year patients’ survival rate was not significantly different between the groups (Figure 2C).

**TABLE 1:** Cohort characteristics.

Characteristics	PDRI					*p*-value
	Q1–1.24 (*n* = 73)	Q2 1.24–1.69 (*n* = 75)	Q3 1.69–2.12 (*n* = 74)	Q4 2.12–2.64 (*n* = 75)	Q5 2.64+ (*n* = 74)	
Recipient factors						
Age (years)	43.8 ± 6.8	43.9 ± 9.2	44.3 ± 7.0	43.3 ± 7.8	45.8 ± 8.1	0.34
Sex (female), n (%)	46 (63.0)	43 (57.3)	49 (66.2)	51 (68.0)	42 (56.8)	0.51
Height (cm)	161.4 ± 9.5	161.3 ± 8.1	160.1 ± 7.4	160.5 ± 7.8	161.2 ± 7.8	0.85
BMI (kg/m^2^)	20.7 ± 2.6	20.7 ± 2.7	21.2 ± 2.7	21.2 ± 2.8	20.5 ± 2.7	0.41
Duration of diabetes (years)	27.3 ± 7.7	29.1 ± 7.8	28.0 ± 8.8	28.0 ± 8.6	28.8 ± 7.4	0.69
Preoperation dialysis, n (%)	61 (83.6)	63 (84.0)	61 (82.4)	61 (81.3)	68 (91.9)	0.36
Duration of dialysis (years)	6.1 ± 5.3	6.6 ± 5.9	6.3 ± 5.8	6.0 ± 5.5	7.1 ± 5.6	0.79
Donor factors						
Age (years)	22.3 ± 5.4	30.9 ± 10.5	42.5 ± 6.5	48.5 ± 5.4	57.4 ± 5.8	<0.001
Sex (female), n (%)	26 (36.1)	29 (38.7)	32 (43.2)	35 (46.7)	39 (52.7)	0.27
Height (cm)	166.6 ± 11.0	161.7 ± 17.1	164.1 ± 8.7	165.4 ± 8.5	159.7 ± 7.9	<0.01
BMI (kg/m^2^)	21.5 ± 3.4	20.5 ± 3.4	22.0 ± 3.4	22.9 ± 3.8	22.5 ± 3.1	<0.001
HbA1c (%)	5.4 ± 0.3	5.3 ± 0.3	5.4 ± 0.4	5.5 ± 0.3	5.6 ± 0.5	<0.001
Cause of death, n (%)						<0.001
CVA	6 (8.2)	20 (26.7)	39 (52.7)	57 (76.0)	65 (87.8)	
Anoxia	28 (38.4)	20 (26.7)	12 (16.2)	9 (12.0)	3 (4.1)	
Trauma	28 (38.4)	20 (26.7)	11 (14.9)	7 (9.3)	4 (5.4)	
Other	11 (15.1)	15 (20.0)	12 (16.2)	2 (2.7)	2 (2.7)	
CPR	41 (56.2)	39 (52.0)	44 (59.5)	26 (34.7)	27 (36.5)	<0.01
SCr (mg/dL)	0.78 ± 0.50	1.14 ± 1.75	1.04 ± 1.08	1.34 ± 1.56	1.23 ± 1.26	0.10
PDRI	1.06 ± 0.09	1.46 ± 0.13	1.89 ± 0.13	2.39 ± 0.15	3.25 ± 0.53	<0.001
Operative factors						
Era						0.28
2001–2010	11 (15.1)	18 (24.0)	17 (23.0)	23 (30.7)	17 (23.0)	
2011–2019	62 (84.9)	57 (76.0)	57 (77.0)	52 (69.3)	57 (77.0)	
Transplantation type						0.03
SPK	57 (78.1)	63 (84.0)	58 (78.4)	62 (82.9)	68 (91.9)	
PAK	16 (21.9)	9 (12.0)	13 (17.6)	7 (9.3)	4 (5.4)	
PTA	0 (0.0)	3 (4.0)	3 (4.1)	6 (8.0)	2 (2.7)	
TIT of the pancreas graft (h)	11.9 ± 2.3	11.9 ± 2.7	11.8 ± 3.0	12.6 ± 3.0	13.3 ± 2.8	<0.01
Graft position (Peritoneal/retroperitoneal)	42/31	51/24	45/28	60/15	58/16	0.01
Ductal management (ED/BD)	62/11	66/9	64/10	65/10	68/6	0.76
Systemic/portal drainage	72/1	74/1	72/2	72/3	74/0	0.44
Carrel patch/Y graft	64/9	63/12	68/6	59/16	66/8	0.16
GDA extension, n (%)	32 (43.8)	34 (45.3)	39 (52.7)	46 (61.3)	46 (62.2)	0.07
Portal vein extension, n (%)	14 (19.2)	18 (24.0)	16 (21.6)	20 (26.7)	16 (21.6)	0.85

Values represent n, n (%), or the mean ± standard deviation.

Abbreviations: PDRI, pancreas donor risk index; BMI, body mass index; HbA1c, hemoglobin A1c; CVA, cerebrovascular accident; CPR, cardiopulmonary resuscitation; SCr, serum creatinine; SPK, simultaneous pancreas and kidney transplantation; PAK, pancreas transplantation after kidney transplantation; PTA, pancreas transplantation alone; TIT, total ischemic time; ED, enteric drainage; BD, bladder drainage; GDA, gastroduodenal artery.

### Comparison Between Japanese Donors and Reference Donors

Axelrod et al. defined the following as reference donors with a PDRI = 1: male sex, 28 years old, non-black, non-Asian, BMI of 24 kg/m^2^, height of 173 cm, cause of death is not CVA, total ischemic time of 12 h for the pancreas graft, no donation after circulatory death, and creatinine concentrations < 2.5 mg/dL [10]. Table 2 shows the features of the average donor in Japan. The median value of each variable was adopted for continuous variables, and factors that accounted for a larger proportion were adopted as categorical variables. As a result, the PDRI was 1.38 times higher for those aged 40.4 years, 1.17 times higher for Asians, 1.06 times higher for a height of 163 cm, and 1.23 times higher for death due to CVA. The incorporation of these factors increased the PDRI value of the average Japanese donor, with an average PDRI value as high as 2.01.

**TABLE 2:** Comparison between Japanese donors and reference donors.

Donor characteristics	Reference donor (PDRI = 1.00)	Japanese donor	Fluctuation in the PDRI
Sex	Male	Male	1.00
Age (years)	28	40.4	1.38
Black race	No	No	1.00
Asian race	No	Yes	1.17
BMI (kg/m^2^)	24	21.9	1.00
Height (cm)	173	163	1.06
Cause of death: CVA/stroke	No	Yes	1.23
Cause of death: CVA/stroke in PAK	No	Yes	0.93
Pancreas preservation time (h)	12	12.3	1.00
DCD	No	No	1.00
SCr > 2.5 (mg/dL)	No	No	1.00

Abbreviations: PDRI, pancreas donor risk index; BMI, body mass index; CVA, cerebrovascular accident; PAK, pancreas transplantation after kidney transplantation; DCD, donation after circulatory death; SCr, serum creatinine.

### Univariate and Multivariate Analyses of Associations of Various Factors With 1-Year Pancreas Graft Failure

**FIGURE 3:**
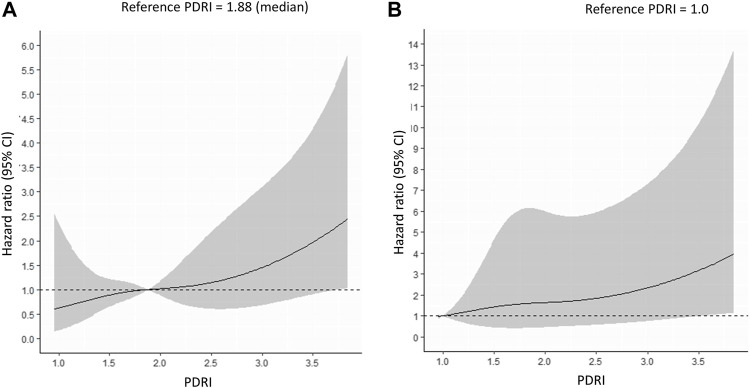
Multivariable-adjusted restricted cubic spline plots of the hazard ratio for 1-year pancreas graft failure. The solid line represents the hazard ratio, and the gray area represents the 95% CI. **(A)** Reference PDRI = 1.88 (median). **(B)** Reference PDRI = 1.0. PDRI, pancreas donor risk index; CI, confidence interval.

A Cox proportional hazards model was used to identify the factors associated with 1-year pancreas graft failure. The univariate analysis showed that the PDRI, donor HbA1c values, cause of death, PAK, and PTA were independent factors that significantly predicted 1-year pancreas graft survival (Table 3). The multivariate analysis excluding the cause of death and PAK included in the PDRI formula showed that the PDRI, donor HbA1c values, and PTA were significant independent factors that predicted 1-year pancreas graft survival (Table 3). A continuous multivariable-adjusted association between the PDRI and 1-year pancreas graft failure was also shown by a restricted cubic spline curve. A median PDRI value of 1.88 and a PDRI of 1.00 were chosen as the reference for each spline plot. The spline curve analysis showed that the PDRI was incrementally associated with an increased risk of 1-year graft failure (Figures 3A,B).

**TABLE 3:** Univariate and multivariate analyses of associations of various factors with 1-year pancreas graft failure.

Coefficient variable	Univariate			Multivariate		
	HR	95% CI	*p*-value	HR	95% CI	*p*-value
Recipient factors						
Age	1.01	0.97–1.05	0.80			
Sex (female)	1.45	0.74–2.84	0.29			
Height	0.97	0.93–1.01	0.08			
BMI	1.05	0.94–1.17	0.41			
Duration of diabetes	1.02	0.98–1.06	0.34			
Duration of dialysis	1.03	0.98–1.09	0.24			
Donor factors						
PDRI, per 0.1	1.05	1.02–1.09	<0.01	1.05	1.01–1.09	0.01
Age	1.02	1.00–1.05	0.05			
Sex (female)	0.96	0.51–1.79	0.89			
Height	1.00	0.97–1.02	0.76			
BMI	1.05	0.96–1.14	0.30			
HbA1c, per 0.1%	1.10	1.03–1.17	<0.01	1.08	1.01–1.15	0.03
Cause of death (CVA)	2.09	1.08–4.05	0.03			
CPR	1.09	0.59–2.03	0.78			
SCr	1.04	0.84–1.29	0.72			
Operative factors						
Era						
2001–2010	0.69	0.31–1.56	0.37			
2010–2019	1.45	0.64–3.27	0.37			
Transplantation type						
SPK	0.60	0.27–1.30	0.19			
PAK	2.39	1.23–4.63	<0.01			
PTA	3.63	1.42–9.26	<0.01	3.65	1.43–9.35	<0.01
TIT	1.07	0.96–1.19	0.20			
GDA reconstruction	0.79	0.42–1.47	0.45			
Portal vein extension	1.13	0.55–2.32	0.73			

Abbreviations: HR, hazard ratio; CI, confidence interval; BMI, body mass index; PDRI, pancreas donor risk index; HbA1c, hemoglobin A1c; CVA, cerebrovascular accident; CPR, cardiopulmonary resuscitation; SCr, serum creatinine; SPK, simultaneous pancreas and kidney transplantation; PAK, pancreas transplantation after kidney transplantation; PTA, pancreas transplantation alone; TIT, total ischemic time; GDA, gastroduodenal artery.

### Transplant Outcomes From Donors With an Extremely High PDRI

The range of PDRI values evaluated by Axelrod et al. ranged from 0.64 to 2.86 [10], and results from donors with a PDRI > 2.86 have not been validated. Therefore, we focused our study on 56 patients with a PDRI ≥ 2.87. The patients were divided into two groups according to a PDRI of 2.87. There were significant differences in the PDRI (*p* < 0.001), age (*p* < 0.001), height (*p* < 0.01), BMI (*p* < 0.001), HbA1c level (*p* < 0.01), death at CVA (*p* < 0.001), and PAK (*p* = 0.03) between the two groups (Supplementary Table S1). When we compared the 1-year pancreas graft survival rate among the groups, the group with a PDRI ≥ 2.87 had a significantly lower survival rate than the group with a PDRI < 2.87 (78.4% vs. 91.0%) (*p* < 0.01, Supplementary Figure S1). In the group with a PDRI ≥ 2.87, 16 cases of graft failure were observed during the entire observation period (19.5 years). Additionally, 12 of the 16 cases showed graft failure within 1 year. The causes of the 12 graft failures were thrombosis in 8 patients, rejections in 2, duodenal perforation in 1, and unknown in 1. Furthermore, 8 of the 12 patients had graft failure within 1 month, and the reason for all of these graft failures was graft thrombosis.

## Discussion

In Japan, pancreas transplants from expanded criteria donors are frequently performed owing to the unique shortage of brain-dead donors. In this study, the mean and median PDRI values were 2.01 and 1.88, respectively. The reason for this finding is that the donors were older, and the cause of death was often cerebrovascular disease (Table 2). These data are clearly higher than those reported in Poland with a mean PDRI of 0.96 [12], in Netherlands with a median PDRI of 1.24 [14], in Spain with a mean PDRI of 1.08,^16^ in the UK with a median PDRI of 1.30,^11^ in Germany with a median PDRI of 1.30 [13], and in Norway with a median PDRI of 0.93 (Table 4) [17]. Despite the high number of expanded criteria donors in our study, the short- and long-term graft survival rates were acceptable (Figures 2A,B), and the patients’ survival rates were also satisfactory (Figure 2C). These results are comparable to those in populations with a low PDRI [10–13, 16, 17] and in the United States [23]. This finding suggests that many donors with a high PDRI potentially have favorable outcomes.

**TABLE 4:** List of PDRI data from various national registries.

Study	Country	Total sample	Range of PDRIs	Mean PDRI	Median PDRI
Axelrod DA et al. [10]	United States	9,401	0.64–2.86	NA	1.00
Mittal S et al. [11]	United Kingdom	1,021	0.49–3.40	NA	1.30
Śmigielska K et al. [12]	Poland	407	0.59–1.33	0.96	NA
Ayami MS et al. [13]	Germany	327	0.54–2.40	NA	1.30
Blok JJ et al. [14]	Netherlands	349	0.68–2.31	NA	1.24
Franz C et al. [15]	Germany	108	0.96–1.38 (IQR)	NA	1.12
Salamanca-Bustos JJ et al. [16]	Spain	126	0.70–2.00	1.08	NA
Kjøsen G et al. [17]	Norway	344	0.58–2.41	NA	0.93
Mittal S et al. [18]	United Kingdom	90	0.69–2.74	NA	1.73
Present study	Japan	371	0.87–5.03	2.01	1.88

Abbreviations: PDRI, pancreas donor risk index; NA, not available; IQR, interquartile range.

In the multivariate analysis of factors involved in the 1-year graft prognosis, the PDRI, donor HbA1c levels, and PTA were prognostic factors. This analysis confirmed the validity of evaluating the PDRI using the Japanese data. There have been two types of reports on the effectiveness of PDRI as a prognostic factor. Some reports showed that the PDRI was effective [12–14], whereas others showed that the PDRI was not effective [15–17], which may be due to racial differences. Some studies reported that the PDRI was only effective in simultaneous pancreas and kidney transplantation only [11–18]. Our results suggest that although the PDRI is a prognostic factor, even donors with a high PDRI can have acceptable outcomes.

Increasing the donor pool is not a problem that can be accomplished in the short term, and donors with high PDRIs must also be used. However, the acceptable range of the PDRI must be discussed. In this study, as shown by the spline curves in Figures 3A,B, the HR increased as the PDRI increased. We found lower short- and long-term graft survival rates in Q5 with a PDRI of 2.64 or higher (Figures 2A,B). The increase in HR was steep from a PDRI of 2.64, and this value was proposed as the cutoff value (Figures 3A,B). The mean donor age for Q5 was 57.4 years (Table 1), which may be considered as a cutoff value for donor age.

In the study by Axelrod et al., PDRI values were validated only up to 2.87 [10]. In this study, 56 patients had a PDRI > 2.87. We found that the group with a PDRI < 2.87 had a better graft prognosis, but the group with a PDRI ≥ 2.87 also had an acceptable 1-year graft survival rate of 78.4%. However, the significantly low graft survival rate cannot be overlooked and should be limited to older recipients or patients who cannot wait any longer because of frequent hypoglycemic attacks.

A high rate of thrombosis occurs in transplants from donors with a high PDRI, which leads to graft failure. Donor risk factors for thrombosis are age [23–26], cerebrovascular cause of death, and a high BMI [26–28]. With regard to preservation factors, the total ischemic time has a considerable effect on graft failure due to thrombosis [29]. These factors are also components of the PDRI, and the results are congruent. Pancreas transplants from donors with an extremely high PDRI have a high incidence of thrombosis, resulting in early graft failure within 1 month. However, once this period is exceeded, stable results are obtained. Therefore, the use of anticoagulants, such as heparin, is left to the discretion of each institution in Japan, but the use of anticoagulants is strongly recommended in cases of an extremely high PDRI.

A strength of this study is that, to the best of our knowledge, this is the first report on the evaluation of the PDRI against a background of data from a large number of expanded criteria donors. We were able to show the results of patients with extremely high PDRI values, which have rarely been previously verified. Additionally, external evaluation of the PDRI has mainly been conducted in Western populations, and whether the PDRI is effective in Asian populations is unknown [30]. The present study shows an association between the PDRI and prognosis in the Japanese population. This finding suggests that the PDRI can be used as a tool for a pre-procurement scoring system even in the Asian population. However, notably, the range of the PDRI is different from that in the Western population. Other limitations of this study are that the number of cases was not large enough and it was a retrospective study. To validate the effectiveness of the PDRI, we evaluated the 1-year pancreas graft outcomes, which are affected not only by donor factors, but also by other factors (e.g., recipient factors, rejection, and recurrence of type 1 diabetes mellitus). However, the involvement of these factors cannot be ruled out completely. Regarding generalizability, all patients were from Japanese facilities and all patients were Japanese nationals. There is a lack of validation in other Asian countries. Although all transplants were performed at specialist-certified centers, surgeon-related factors may have contributed to the outcomes. Additionally, the study period was extended over almost two decades. The mean PDRI value in patients in 2001–2010 was 2.11 ± 0.76 and that in 2011–2019 was 1.98 ± 0.81 (data not shown in the text), which indicates that donor indications have become more rigorous over time. Advances in pharmacology, technology, and surgical methods during this period could have biased the results toward more recent cases, and older cases may no longer be representative of state-of-the-art situations.

In conclusion, the PDRI is an effective evaluation tool for pancreas transplantation in Japan. Pancreas transplantation from donors with a high PDRI can be performed with acceptable results as an alternative until the donor pool is increased. However, the early development of thrombosis should be noted in cases of an extremely high PDRI.

## Data Availability

The data that support the findings of this study are available from the Japan Society for Transplantation but restrictions apply to the availability of these data, which were used under license for the current study, and so are not publicly available. Requests to access the datasets should be directed to The Japan Society for Transplantation, http://www.asas.or.jp/jst/.

## Supplementary Material

Supplementary Figure S1 Kaplan–Meier curve for comparison of the group with a PDRI < 2.87 and the group with a PDRI ≥ 2.87, with a cutoff value of 2.87.

## Abbreviations

BMI, body mass index; CI, confidence interval; CPR, cardiopulmonary resuscitation; CVA, cerebrovascular accident; GDA, gastroduodenal artery; HbA1c, hemoglobin A1c; HR, hazard ratio; PAK, pancreas transplantation after kidney transplantation; PDRI, pancreas donor risk index; SCr, serum creatinine; SPK, simultaneous pancreas and kidney transplantation; TIT, total ischemic time; PTA, pancreas transplantation alone.

## References

[1] Rayhill SC D’Alessandro AM Odorico JS Knechtle SJ Pirsch JD Heisey DM Simultaneous Pancreas-Kidney Transplantation and Living Related Donor Renal Transplantation in Patients with Diabetes: Is There a Difference in Survival? Ann Surg (2000) 231:417–23. 10.1097/00000658-200003000-00015 10714635 PMC1421013

[2] Sung RS Zhang M Schaubel DE Shu X Magee JC. A Reassessment of the Survival Advantage of Simultaneous Kidney-Pancreas versus Kidney-Alone Transplantation. Transplantation (2015) 99:1900–6. 10.1097/TP.0000000000000663 25757212 PMC4548542

[3] Martins LS Outerelo C Malheiro J Fonseca IM Henriques AC Dias LS Health-related Quality of Life May Improve after Transplantation in Pancreas-Kidney Recipients. Clin Transpl (2015) 29:242–51. 10.1111/ctr.12511 25581297

[4] Rajkumar T Mazid S Vucak-Dzumhur M Sykes TM Elder GJ. Health-related Quality of Life Following Kidney and Simultaneous Pancreas Kidney Transplantation. Nephrology (2019) 24:975–82. 10.1111/nep.13523 30393905

[5] Kaku K Kitada H Noguchi H Kurihara K Kawanami S Nakamura U Living Donor Kidney Transplantation Preceding Pancreas Transplantation Reduces Mortality in Type 1 Diabetics with End-Stage Renal Disease. Transpl Proc (2015) 47:733–7. 10.1016/j.transproceed.2014.12.048 25891721

[6] Gruessner RW Sutherland DE Gruessner AC. Mortality Assessment for Pancreas Transplants. Am J Transpl (2004) 4:2018–26. 10.1111/j.1600-6143.2004.00667.x 15575904

[7] Tomimaru Y Eguchi H Doki Y Ito T Kenmochi T. Current State of Pancreas Transplantation in Japan Based on the Nationwide Registry. Ann Gastroenterol Surg (2021) 5:494–501. 10.1002/ags3.12423 34337298 PMC8316743

[8] Tomimaru Y Kobayashi S Ito T Iwagami Y Yamada D Akita H Clinical Impact of Pancreas Donor Age on Outcomes Following Pancreas Transplantation: Analysis of a Nationwide Registry in Japan. Pancreatology (2021) 21:473–9. 10.1016/j.pan.2021.01.002 33461932

[9] Ito T Kenmochi T Aida N Kurihara K Asaoka T Ito T. Are the Outcomes of Japanese Pancreas Transplantation Utilizing Extended-Criteria Donors Acceptable? A Propensity Score Matching Analysis for Donors <50 or ≥50 Years Old. Transpl Int (2020) 33:1046–60. 10.1111/tri.13636 32394519

[10] Axelrod DA Sung RS Meyer KH Wolfe RA Kaufman DB. Systematic Evaluation of Pancreas Allograft Quality, Outcomes and Geographic Variation in Utilization. Am J Transpl (2010) 10:837–45. 10.1111/j.1600-6143.2009.02996.x 20121753

[11] Mittal S Lee FJ Bradbury L Collett D Reddy S Sinha S Validation of the Pancreas Donor Risk Index for Use in a UK Population. Transpl Int (2015) 28:1028–33. 10.1111/tri.12563 25789920

[12] Śmigielska K Skrzypek P Czerwiński J Michalak G Durlik M Grochowiecki T Usefulness of Pancreas Donor Risk index and Pre-procurement Pancreas Allocation Suitability Score: Results of the Polish National Study. Ann Transpl (2018) 23:360–3. 10.12659/AOT.909654 PMC6248280 29798972

[13] Ayami MS Grzella S Kykalos S Viebahn R Schenker P. Pancreas Donor Risk index but Not Pre-procurement Pancreas Allocation Suitability Score Predicts Pancreas Graft Survival: a Cohort Study from a Large German Pancreas Transplantation center. Ann Transpl (2018) 23:434–41. 10.12659/AOT.910014 PMC6248050 29941863

[14] Blok JJ Kopp WH Verhagen MJ Schaapherder AF de Fijter JW Putter H The Value of PDRI and P-PASS as Predictors of Outcome after Pancreas Transplantation in a Large European Pancreas Transplantation center. Pancreas (2016) 45:331–6. 10.1097/MPA.0000000000000485 26474435

[15] Franz C Görtz M Wührl M Kulu Y Hoffmann K Hackert T The Role of Pre-procurement Pancreas Suitability Score (P-PASS) and Pancreas Donor Risk index (PDRI) in the Outcome of Simultaneous Pancreas and Kidney or Pancreas after Kidney Transplantation. Ann Transpl (2019) 24:439–45. 10.12659/AOT.915852 PMC6681688 31346153

[16] Salamanca-Bustos JJ Campos-Hernandez JP Sánchez-Hidalgo JM Arjona-Sánchez A Sánchez-González A Arenas-Bonilla AJ Validation of the Pancreatic Donor Risk index in Simultaneous Pancreas-Kidney Transplantation Performed in Córdoba Hospital from 2000 to 2015. Transpl Proc (2016) 48:3037–9. 10.1016/j.transproceed.2016.07.049 27932141

[17] Kjøsen G Horneland R Nordheim E Aandahl EM Line P-D Rydenfelt K Validating the US Pancreas Donor Risk index in a Norwegian Population, a Retrospective Observational Study. Scand J Gastroenterol (2022) 57:345–51. 10.1080/00365521.2021.2012590 35130456

[18] Mittal S Sharples E Lee F Reddy S Sinha S Friend P App to Reality: Snapshot Validation of the US Pancreas Donor Risk Index in a UK center. J Surg Res (2013) 183:841–5. 10.1016/j.jss.2013.03.098 23623570

[19] Mensink JW de Vries KM Huurman VAL Pol RA Alwayn IPJ Braat AE. Risk Analysis of Extended Pancreas Donor Selection Criteria. Pancreatology (2019) 19:994–9. 10.1016/j.pan.2019.08.010 31495709

[20] Cornateanu SM O'Neill S Dholakia S Counter CJ Sherif AE Casey JJ Pancreas Utilization Rates in the UK - an 11-year Analysis. Transpl Int (2021) 34:1306–18. 10.1111/tri.13876 33794037

[21] Alhamad T Malone AF Lentine KL Brennan DC Wellen J Chang S-H Selected Mildly Obese Donors Can Be Used Safely in Simultaneous Pancreas and Kidney Transplantation. Transplantation (2017) 101:1159–66. 10.1097/TP.0000000000001303 27428713

[22] Kanda Y. Investigation of the Freely Available Easy-To-Use Software ‘EZR’ for Medical Statistics. Bone Marrow Transpl (2013) 48:452–8. 10.1038/bmt.2012.244 PMC3590441 23208313

[23] Humar A Kandaswamy R Granger D Gruessner AC Sutherland DE. Decreased Surgical Risks of Pancreas Transplantation in the Modern Era. Ann Surg (2000) 231:269–75. 10.1097/00000658-200002000-00017 10674620 PMC1420996

[24] Gruessner AC Gruessner RW. Pancreas Transplantation of US and Non‐US Cases from 2005 to 2014 as Reported to the United Network for Organ Sharing (UNOS) and the International Pancreas Transplant Registry (IPTR). Rev Diabet Stud (2016) 13:35–58. 10.1900/RDS.2016.13.35 26982345 PMC5291181

[25] Troppmann C Gruessner AC Benedetti E Papalois BE Dunn DL Najarian JS Vascular Graft Thrombosis after Pancreatic Transplantation: Univariate and Multivariate Operative and Nonoperative Risk Factor Analysis. J Am Coll Surg (1996) 182:285–316. 8605554

[26] Gruessner RW Sutherland DE Troppmann C Benedetti E Hakim N Dunn DL The Surgical Risk of Pancreas Transplantation in the Cyclosporine Era: an Overview. J Am Coll Surg (1997) 185:128–44. 10.1016/s1072-7515(01)00895-x 9249080

[27] Humar A Ramcharan T Kandaswamy R Gruessner RWG Gruessner AC Sutherland DER. Technical Failures after Pancreas Transplants: Why Grafts Fail and the Risk Factors—A Multivariate Analysis. Transplantation (2004) 78:1188–92. 10.1097/01.tp.0000137198.09182.a2 15502718

[28] Humar A Ramcharan T Kandaswamy R Gruessner RWG Gruessner AG Sutherland DER. The Impact of Donor Obesity on Outcomes after Cadaver Pancreas Transplants. Am J Transpl (2004) 4:605–10. 10.1111/j.1600-6143.2004.00381.x 15023153

[29] Grewal HP Garland L Novak K Gaber L Tolley EA Gaber AO. Risk Factors for post Implantation Pancreatitis and Pancreatic Thrombosis in Pancreas Transplant Recipients. Transplantation (1993) 56:609–12. 10.1097/00007890-199309000-00021 8212156

[30] Han D-J Kenmochi T Shyr Y-M. Pancreas Transplantation – the Asian Experience- A Registry Report. Singapore: Springer (2022).

