# Supplementary material for: Utilization of the Pancreas From Donors With an Extremely High Pancreas Donor Risk Index: Report of the National Registry of Pancreas Transplantation
Source: Transpl Int. 2023 May 17;36:11132. doi: 10.3389/ti.2023.11132 (PMC10229828; doi:10.3389/ti.2023.11132)
Supplement: Supplementary file 1 [file Table1.docx]

**Table S1. Cohort characteristics with extremely high Pancreas Donor Risk Index**

| Characteristic | PDRI ≥ 2.87  N=56 | PDRI < 2.87  N=315 | *P* value |
| --- | --- | --- | --- |
| **Donor factors** |  |  |  |
| PDRI | 3.41± 0.51 | 1.76 ± 0.55 | < 0.001 |
| Age (years) | 58.7 ± 5.3 | 37.1 ± 12.8 | < 0.001 |
| Sex (female), n (%) | 29 (51.8) | 132 (63.8) | 0.18 |
| Height (cm) | 159.1 ± 7.4 | 164.2 ± 11.8 | < 0.01 |
| BMI (kg/m^2^) | 22.4 ± 3.1 | 21.8 ± 3.6 | < 0.001 |
| HbA1c (%) | 5.6 ± 0.5 | 5.4 ± 0.3 | < 0.01 |
| Cause of death, n (%) |  |  |  |
| CVA | 51 (91.1) | 136 (43.2) | < 0.001 |
| Anoxia | 1 (1.8) | 71 (22.5) | < 0.001 |
| Trauma | 2(3.6) | 68 (21.6) | < 0.01 |
| Other | 2 (3.6) | 40 (12.7) | < 0.05 |
| CPR, n (%) | 20 (35.7) | 157 (49.8) | 0.05 |
| Operation Type, n (%) |  |  |  |
| SPK | 52 (92.9) | 256 (81.3) | 0.15 |
| PAK | 4 (7.1) | 45 (14.3) | 0.03 |
| PTA | 0 | 14 (4.4) | 0.11 |

Values represent n (%) or mean ± standard deviation.

Abbreviations: PDRI, Pancreas Donor Risk Index; BMI, body mass index; HbA1c, hemoglobin A1c; CVA, cerebrovascular accident; CPR, cardiopulmonary resuscitation; SPK, simultaneous pancreas and kidney transplantation; PAK, pancreas transplantation after kidney transplantation; PTA, pancreas transplantation alone.
