# Supplementary figures and images for: Utilization of the Pancreas From Donors With an Extremely High Pancreas Donor Risk Index: Report of the National Registry of Pancreas Transplantation
Source: Transpl Int. 2023 May 17;36:11132. doi: 10.3389/ti.2023.11132 (PMC10229828; doi:10.3389/ti.2023.11132)

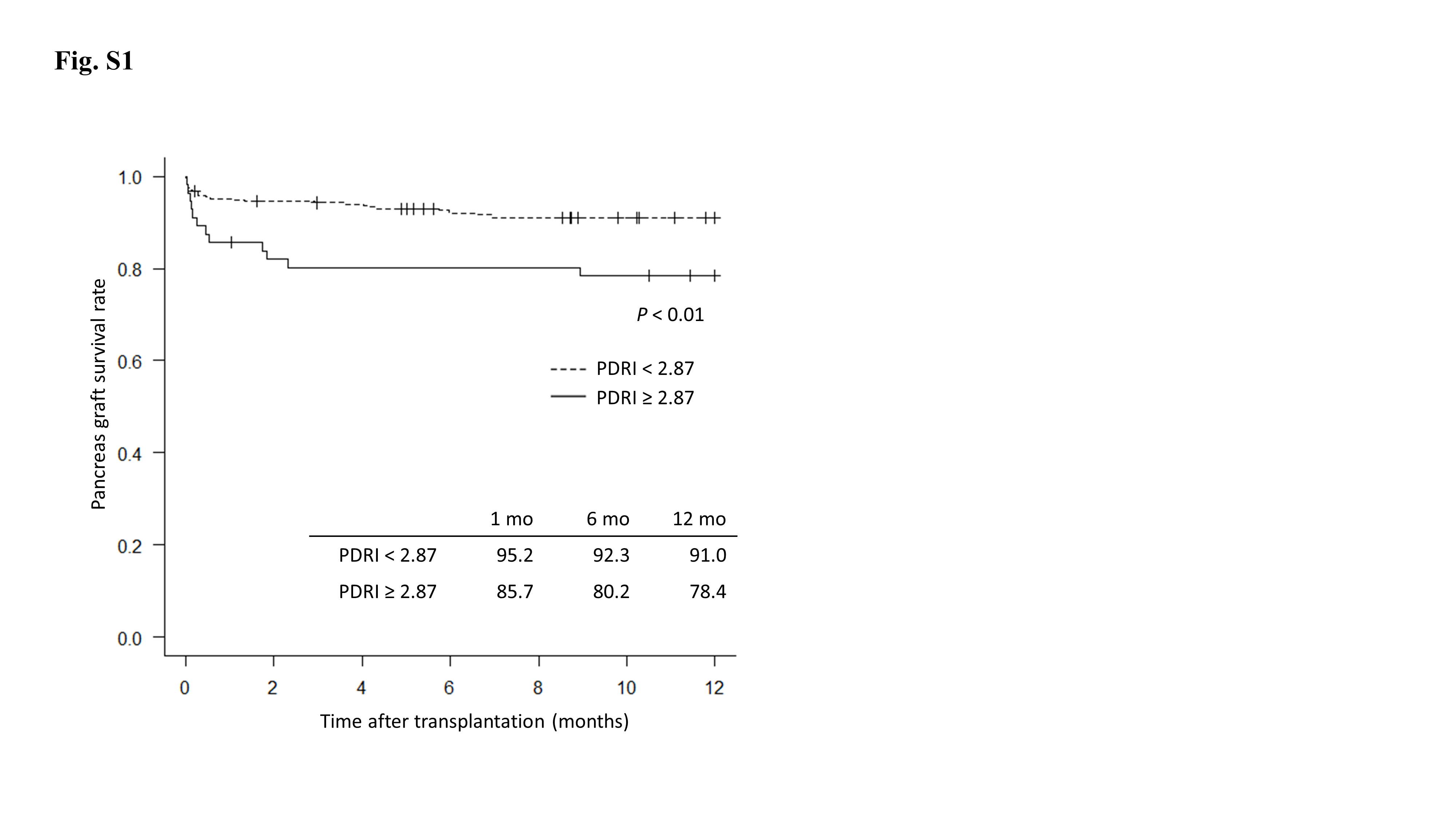

Supplement: Supplementary file 2 [file Image1.TIF]
